# Autologous Antibody Capture to Enrich Immunogenic Viruses for Viral Discovery

**DOI:** 10.1371/journal.pone.0078454

**Published:** 2013-11-04

**Authors:** Bas B. Oude Munnink, Seyed Mohammad Jazaeri Farsani, Martin Deijs, Jiri Jonkers, Joost T. P. Verhoeven, Margareta Ieven, Herman Goossens, Menno D. de Jong, Ben Berkhout, Katherine Loens, Paul Kellam, Margreet Bakker, Marta Canuti, Matthew Cotten, Lia van der Hoek

**Affiliations:** 1 Laboratory of Experimental Virology, Department of Medical Microbiology, Center for Infection and Immunity Amsterdam (CINIMA), Academic Medical Center of the University of Amsterdam, Amsterdam, The Netherlands; 2 Tehran University of Medical Sciences, Tehran, Iran; 3 Department of Medical Microbiology, Vaccine and Infectious Disease Institute, University Hospital, Antwerp, Belgium; 4 Wellcome Trust Sanger Institute, Hinxton, United Kingdom; Kantonal Hospital St. Gallen, Switzerland

## Abstract

Discovery of new viruses has been boosted by novel deep sequencing technologies. Currently, many viruses can be identified by sequencing without knowledge of the pathogenicity of the virus. However, attributing the presence of a virus in patient material to a disease in the patient can be a challenge. One approach to meet this challenge is identification of viral sequences based on enrichment by autologous patient antibody capture. This method facilitates identification of viruses that have provoked an immune response within the patient and may increase the sensitivity of the current virus discovery techniques. To demonstrate the utility of this method, virus discovery deep sequencing (VIDISCA-454) was performed on clinical samples from 19 patients: 13 with a known respiratory viral infection and 6 with a known gastrointestinal viral infection. Patient sera was collected from one to several months after the acute infection phase. Input and antibody capture material was sequenced and enrichment was assessed. In 18 of the 19 patients, viral reads from immunogenic viruses were enriched by antibody capture (ranging between 1.5x to 343x in respiratory material, and 1.4x to 53x in stool). Enriched reads were also determined in an identity independent manner by using a novel algorithm Xcompare. In 16 of the 19 patients, 21% to 100% of the enriched reads were derived from infecting viruses. In conclusion, the technique provides a novel approach to specifically identify immunogenic viral sequences among the bulk of sequences which are usually encountered during virus discovery metagenomics.

## Introduction

Virus infections are a continuous threat to the human population; e.g. HIV, hepatitis viruses, and influenza viruses constitute a large proportion of morbidity and mortality each year. Apart from infection with well-described viruses, outbreaks with previously undescribed viruses occur regularly (e.g. SARS-CoV, MERS-CoV) [1]–[4]. Furthermore, respiratory tract infections and diarrhoea in young children or immunocompromised persons often test negative for known viruses, and could very well be caused by yet unknown pathogens.

Discovery of new viruses in the last decade has been boosted by large improvements in sequencing technology. These methods form the basis for improved virus discovery processes capable of generating 10e5–10e7 sequence reads directly from a clinical sample. A virus discovery method to amplify RNA and DNA virus sequences directly in patient material (VIDISCA-454) without prior knowledge of the viral genome sequence has been developed [5]. The resulting DNA library is then subjected to Roche-454 next generation sequencing and this method has been successfully used to identify human coronavirus NL63 [6], a novel HIV-1 subtype [7], and 2 novel parvoviruses in bats [8].

One limitation of the current technique is that a substantial amount of non-viral RNA and DNA derived from the host or from other agents in the sample can dominate the resulting sequences. Especially in respiratory samples, ribosomal RNA is massively present, over 80% of all sequence reads derived from a clinical sample originate from this material [9]. Sequence reads from fecal samples can be dominated by bacterial or dietary components. A method for focusing sequencing on immunogenic viruses was sought.

Another limitation of the current techniques is that detection of reads derived from a known virus does not necessarily indicate that this virus is a pathogen. Recently, many new viruses have been identified in human samples without clear association with disease, necessitating further detailed investigations to determine the pathogenicity of the virus [10]–[13].

To facilitate the detection of immunogenic viruses and to reduce the detection of non disease-related viruses (bacteriophages and plant viruses) and host cellular RNA, a technique was developed that uses convalescent serum of the patient to concentrate viruses that have elicited and immune response prior to sequencing. Comparing the sequences derived from input and antibody captured material identifies immunogenic agents and can provide an important first step in identifying a disease-related virus.

## Methods

### Samples

Respiratory samples were collected during the GRACE study. Flocked nasopharyngeal swabs (Copan) were collected in universal transport medium (UTM). In addition, a single nasopharyngeal specimen was obtained at the Academic Medical Center from a patient with an upper respiratory tract infection.

Fecal samples were selected from a sample bank from 196 HIV-1-infected patients with and without diarrhea, aged above 18 who visited the out-patients clinic at the Academic Medical Center in the years 1994–1995**.** Fecal samples were suspended in broth (1:3 dilution, Oxoid nutrient broth no.2, pH 7.5).

### Ethical approval

Ethics review committees in each country approved the study, Cardiff and Southampton (United Kingdom): Southampton & South West Hampshire Research Ethics Committee A; Utrecht (Netherlands) Medisch Ethische Toetsingscommissie Universitair Medisch Centrum Utrecht; Barcelona (Spain) Comitè ètic d'investigació clínica Hospital Clínic de Barcelona; Mataro (Spain): Comitè d'Ètica d'Investigació Clínica (CEIC) del Consorci Sanitari del Maresme; Rotenburg (Germany) Ethik-Kommission der Medizinischen Fakultät der Georg-August-Universität Göttingen, Antwerpen (Belgium): UZ Antwerpen Comité voor Medische Ethiek; Lodz, Szeczecin, and Bialystok (Poland): Komisja Bioetyki Uniwersytetu Medycznego W Lodzi; Milano (Italy) IRCCS Fondazione Cà Granda Policlinico; Jonkoping (Sweden): Regionala etikprövningsnämnden i Linköping; Bratislava (Slovakia): Etika Komisia Bratislavskeho; Gent (Belgium): Ethisch Comité Universitair Ziekenhuis Gent; Nice (France) Comité de Protection des Personnes Sud-Méditerranée II, Hôpital Salvator; Jesenice (Slovenia): Komisija Republike Slovenije za Medicinsko Etiko. Written informed consent was provided by all study participants.

Collection of fecal material was performed in accordance with the ethical principles set out in the declaration of Helsinki and written informed consent has been obtained prior to data collection. The study was approved by the Amsterdam Medical Center institutional medical ethics committee.

### Antibody capture

Respiratory and fecal samples were centrifuged (10,000 g) and 150 µl of the supernatant was mixed with 50µl Dynabeads protein A and G (1∶1, Invitrogen). After 20 minutes incubation, 10 µl of autologous convalescent serum of the patient was added to the mixture (Table 1). After a 20 minutes incubation with continuous shaking at room temperature, samples were washed six times with PBS using a magnetic particle concentrator. Universal transport medium with TURBO™ DNase (2U/µl, Ambion) was added to the antibody-antigen complex and samples were incubated at 37°C for 30 minutes. The complexes were lysed with Boom-lysis buffer L6 and the lysate was used as input for Boom extraction [14], followed by VIDISCA-454 sequencing as described below.

**Table 1:** Collection of serum in month(s) after infection.

Patient ID	ΔT since acute phase	Patient ID	ΔT since acute phase
**Patient 1**	1 month	**Patient 11**	1 month
**Patient 2**	1 month	**Patient 12**	1 month
**Patient 3**	1 month	**Patient 13**	2 months
**Patient 4**	1 month	**Patient 14**	3 months
**Patient 5**	1 month	**Patient 15**	5 months
**Patient 6**	1 month	**Patient 16**	3 months
**Patient 7**	1 month	**Patient 17**	8 months
**Patient 8**	1 month	**Patient 18**	4 months
**Patient 9**	1 month	**Patient 19**	13 months
**Patient 10**	1 month		

### Real time RT-PCR for ribosomal RNA

Real time PCR for ribosomal RNA was performed as described [5] using primer set 5/6 and the rRNA28S_3674 probe. The platinum quantitative PCR Supermix-UDG system (Invitrogen) was used.

### VIDISCA and Roche Titanium-454 sequencing

VIDISCA-454 was performed as previously described [5]. In short, samples were centrifuged for 10 minutes at 10,000 g and the supernatant was treated with DNase. Subsequently, nucleic acids were extracted by the Boom extraction method [14]. rRNA-blocking oligonucleotides were added to prevent amplification of ribosomal RNA and a reverse transcription reaction with Superscript II (Invitrogen) was performed using non-ribosomal random hexamers [15]. Subsequently, second strand DNA synthesis was performed with 5 U of Klenow fragment (Westburg). Double-stranded DNA was purified by phenol/chloroform extraction and ethanol precipitation and digested with Mse I restriction enzyme (New England Biolabs). Adaptors with different Multiplex Identifier sequences (MIDs) were ligated to the digested fragments of the different samples. Next, a PCR with adaptor-binding primers was performed. After purification (Agencourt AMPure XP PCR, Beckman Coulter), the purified DNA was quantified with the Quant-it dsDNA HS Qubit kit (Invitrogen) and diluted to 10^7^ ng/µl. Samples were pooled and Kapa PCR (Kapa Biosystems) was performed to determine the quantity of amplifiable DNA in each pool. Subsequently, the Bioanalyser (hsDNA chip, Agencourt) was used to determine the average nucleotide length of the libraries and the pools were diluted until 10^6^ copies/µl to be used for a titration (DNA:beads ratio of 0.5∶1, 1∶1, 2∶1 and 4∶1) in an emulsion PCR according to the suppliers’ protocol (LIB-A SV emPCR kit). Sequencing was done on a 2 region GS FLX Titanium PicoTiterPlate (70×75) with GS FLX Titanium XLR 70 Sequencing kit (Roche).

### Sequence analysis

Primers, MIDs and ribosomal RNA sequences were trimmed from the reads. Sequences were compared with all available sequences in the nonredundant Genbank database [16] via the BlastN (http://blast.ncbI.nlm.nih.gov/Blast.cgi) tool [17]. The following Blast settings were used: expect threshold: 1000, Match/Mismatch Scores: 1.-1, Gap Costs: Existence: 2 Extension: 1. The blast output was subsequently used to create a taxonomic classification of the reads with Megan software version 4.70.4 [18]. The following settings were used: Min Support: 1, Min Score: 80, and Top Percent 100. The sequences reads are submitted to the European Nucleotide Archive, accession number PRJEB4561.

### Enrichment index

The percentage of viral sequences was calculated by dividing the number of virus derived reads in the sample by the total numbers of reads in the same sample. The enrichment index was calculated by dividing the percentage of viral sequences in the captured sample by the percentage viral sequences in the input sample. A value above 1 indicates antibody capture of the virus, which suggests a immunogenic course of infection.

### Determination of enriched sequences

To identify sequences which are enriched by antibody capture, input and enriched sequences were compared using a Python algorithm *xcompare* (source available on request). The algorithm is comprised of the following steps: the identification of identical or nearly-identical sequences within the input subset was performed by creating a custom BLAST database [19] comprising all the reads derived from the input sample and subsequently performing a BLAST search within this database towards its own sequences. Via this method a list of closely related sequences could be created which were extracted and aligned via MUSCLE 3.8.31 [20], [21] (maximum number of iterations: 1; diagonal optimization enabled) and both consensus sequences and unique sequences were joined into a unique fragment library. In turn, this unique fragment library was converted into a second custom BLAST database, against which the reads obtained from the enriched sample were compared. For the BLAST comparison the following settings were used: E-value threshold 3E-60 for within the input sample library and 3E-25 for between the enriched and input samples library; word size: 11; match/mismatch scores 1/-2; gap existence/extension penalty: 5/2.

The second BLAST analysis yields the percentage of sequence space occupied by each fragment in the original sample (number of reads comprising a fragment, divided by the total number of reads in the input sample library) and the enriched sample (number of sequences in the enriched sample matching to a specific fragment, divided by the total number of reads in the enriched sample library). The ratio between these percentages is calculated and all fragments with a ratio higher than 1.0 were extracted and further analyzed.

## Results

Serum collected a few weeks to a few months after respiratory or gastrointestinal infection generally contains a substantial amount of pathogen-specific immunoglobulin type G (IgGs) with a proportion of these antibodies binding to virus surface exposed epitopes [22]–[24]. These IgGs can be bound to magnetic beads and used to capture a target virus and to separate it from non-viral material (e.g. ribosomes) or non-immunogenic viruses (e.g. plant viruses in stool). After deep sequencing, comparison of reads in the captured material to reads in the input material should reveal virus-specific reads via capture by the antibodies. We tested this strategy in 13 respiratory samples, diagnosed as containing one of the following viruses: human parainfluenza virus 1, 2 and 4, human rhinovirus, human metapneumovirus virus, influenza virus A and B, human respiratory syncytial virus and human coronavirus 229E, OC43 and NL63. We also tested 6 fecal samples containing adenovirus, norovirus, enterovirus and sapovirus. Autologous convalescent sera collected one to a few months after infection was available for all samples (see table 1). Plant viruses and enterobacteriophages - viruses which are frequently present in feces - were used as negative control. These viruses are not known to elicit an immune response and are not expected to be captured by the antibody-bound beads.

### Enrichment index

Of the 13 respiratory samples and the 6 fecal samples, a total of 110,752 reads were obtained from the input material and 93,779 reads were obtained from the antibody captured material with a median read length of 159 nucleotides. For each patient the enrichment index was determined by calculating the ratio between the percentage of viral reads in the captured sample versus the input sample (Table 2).

**Table 2:** Reads of viruses after antibody capture.

Patient ID	Virus	% viral reads in input	% viral reads in capture		Enrichment index^a^
**Patient 1**	**PIV-1**	0.26%	0.38%		1.5
**Patient 2**	**PIV-2**	0.07%	3.6%		54
**Patient 3**	**PIV-4**	0.78%	15%		19
**Patient 4**	**HRV**	42%	77%		1.9
**Patient 5**	**hMPV**	1.4%	9.9%		7.2
**Patient 6**	**NL63**	1.7%	33%		20
**Patient 7**	**RSV**	18%	29%		1.6
**Patient 8**	**Inf B**	0.61%	2.0%		3.3
**Patient 9**	**Inf A**	3.0%	75%		25
**Patient 10**	**OC43**	0.35%	59%		169
**Patient 11**	**NL63**	0.14%	48%		343
**Patient 12**	**229E**	0.55%	13%		23
**Patient 13**	**HRV**	1.4%	4.5%		3.1
**Patient 14**	**Norovirus**	0.16%	3.2%		20
**Patient 14**	**Adenovirus**	3.9%	3.7%		0.9
**Patient 15**	**Norovirus**	8.3%	88%		11
**Patient 16**	**Enterovirus**	0.03%	0.18%		5.2
**Patient 17**	**Sapovirus**	0.08%	0.40%		4.9
**Patient 18**	**Sapovirus**	3.2%	4.5%		1.4
**Patient 18**	**Enterovirus**	1.0%	0.00%		0.00
**Patient 19:**	**Hepatitis B virus**	0.05%	2.50%		53
**Patient 15**	**Cucumber mosaic virus** b	0.36%	0.03%		0.08
**Patient 19:**	**Caudovirales** b	63%	0.62%		0.01

a The enrichment index is calculated by dividing the percentage of virus reads in the captured material by the number of virus reads in the input material.

b Control, non pathogenic viruses.

For all respiratory samples the enrichment index was above 1.0, indicating that in every sample tested the number of viral reads increased after capture. The human coronaviruses OC43, NL63 and 229E were detected with an enrichment index of 169, 343 and 23 respectively. In a second NL63 case (patient 6), the virus was enriched 20-fold. The para-influenza viruses 1, 2 and 4 infections were detected with an enrichment index of 1.5, 54 and 19 respectively. The enrichment index for the metapneumovirus was 7.2, while it was 1.6 for the respiratory syncytial virus. Human rhinovirus infection was observed in two different patients and the enrichment index was 1.9 and 3.1 respectively. Influenza B virus was detected with an enrichment index of 3.3 and influenza A virus with an enrichment index of 25.

Fecal samples were analyzed by the same process. Norovirus infection was observed in two patients and enriched 20-fold and 11-fold. Adenovirus was also detected in a patient with norovirus infection and no enrichment for adenovirus was scored (0.9). Enterovirus reads were detected with an enrichment factor of 5.2 in one case but enterovirus reads were not enriched (enrichment factor of 0.0) in a second case. Viral reads derived from sapovirus were detected in samples from two patients with an enrichment factor of 4.9 and 1.4 respectively. Hepatitis B viral reads were detected in patient 19 with an enrichment factor of 53. As controls, a plant virus (cucumber mosaic virus) and an enterobacteriophage were tested. These viruses had an enrichment index of 0.08 and 0.01 respectively, indicating that there presence was reduced in the captured material.

**Figure 1:**
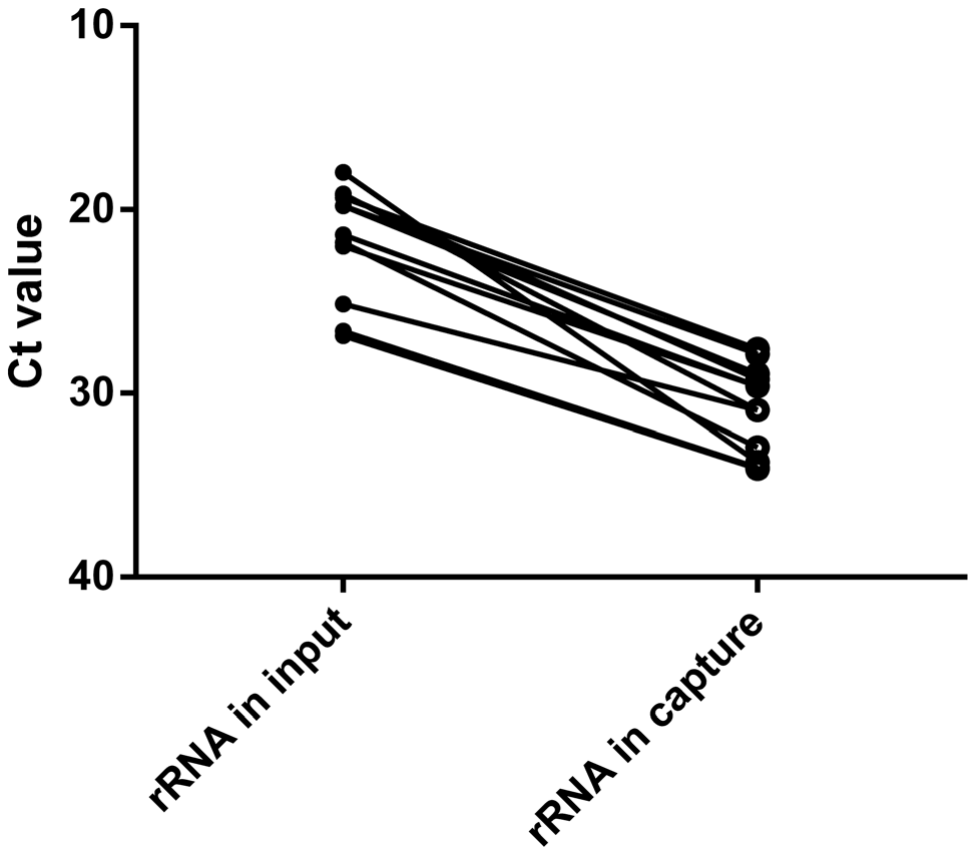
Decrease in ribosomal RNA after antibody capture. Ribosomal RNA was measured in the input material and the captured material. On the Y-axis the Ct value of the real time PCR on the cDNA is shown.

Consistent with capture working as anticipated, reads derived from human ribosomal RNA showed a consistent reduction in the antibody captured fractions with an average decrease of 1,000 fold (a median decrease of 9.6 Ct values, Figure 1). Greater than 90% of the reads showing decreased levels were of ribosomal RNA origin in 11 of the 13 patients. In the two other cases the amount of ribosomal RNA was low in the input material, but commensal bacterial reads were massively present and for these patients a strong decrease (>70%) was observed in the number of bacterial reads (data not shown).

### Identity independent enrichment

The enrichment index shows that viral reads appear more frequently in the antibody-captured material compared to the input. This increase can also be used to identify viruses without the necessity of having sequence similarity to known viruses that can be probed by Blast to search for identity. Since restriction enzyme digestion is part of the protocol, identical fragments of the same size will be generated from identical viruses and this means that the number of these identical fragments should increase if the relative load of the virus increases. Xcompare was used to calculate the frequency distribution between the input and the captured reads (see Materials and Methods). Reads present in higher quantity in the captured samples compared to the input samples were selected. Reads originated from archaea, bacteria or eukaryota were excluded and the remaining reads, (Enriched Analysis Reads, EAR), were further investigated.

EAR values ranged from 0 to 3019 (Table 3). In respiratory samples, almost all EAR were derived from viruses. In patients 3–13, 98–100% of all EAR sequences were of viral origin. Also in fecal samples, virus reads represent the majority of the EAR. In patient 14, 30% of the EAR were derived from norovirus and 50% from adenovirus. In patient 15, all 3019 enriched reads were derived from norovirus. In patient 16 none of the 42 EAR were derived from an enterovirus, and also in patient 18 no enterovirus reads were detected in the EAR as expected since no enterovirus reads were present in the captured reads. Sapovirus sequences were detected in the EAR of patient 17 and 18, 21% and 65% respectively. In patient 19, 62% of the EAR were derived from a hepatitis B virus. The plant virus and the enterobacteriophage were not detected in the EAR of patient 15 and 19.

**Table 3:** Reads of viruses after antibody capture.

Patient ID	Virus	Sequences in the enriched analysis-pool^a^	% Viral sequences
**Patient 1**	**PIV-1**	0	0%
**Patient 2**	**PIV-2**	0	0%
**Patient 3**	**PIV-4**	99	98%
**Patient 4**	**HRV**	1945	100%
**Patient 5**	**hMPV**	65	100%
**Patient 6**	**NL63**	177	100%
**Patient 7**	**RSV**	636	99%
**Patient 8**	**Inf B**	13	100%
**Patient 9**	**Inf A**	1634	100%
**Patient 10**	**OC43**	71	100%
**Patient 11**	**NL63**	249	100%
**Patient 12**	**229E**	15	100%
**Patient 13**	**Rhino A**	124	100%
**Patient 14**	**Norovirus**	349	30%
**Patient 14**	**Adenovirus**	349	50%
**Patient 15**	**Norovirus**	3019	100%
**Patient 16**	**Enterovirus**	121	0.00%
**Patient 17**	**Sapovirus**	42	21%
**Patient 18**	**Sapovirus**	246	65%
**Patient 18**	**Enterovirus**	246	0.00%
**Patient 19:**	**Hepatitis B virus**	21	62%
**Patient 15**	**Cucumber mosaic virus** b	3019	0.00%
**Patient 19:**	**Caudovirales** b	21	0.00%

a Only enriched sequences with the potency to be viral are shown, so no known bacterial, human, fungal, etc. or other sequences are included.

b Control, non pathogenic viruses.

## Discussion

A new method is described that facilitates virus identification by enriching viral material with convalescent autologous patient antibody capture followed by deep sequencing. For all samples tested here, an increase in percentage of viral reads was detected when compared to the input sample. In 9 out of the 21 viruses the amount of viral reads after capture increase 10-fold or more.

By using the Xcompare script, enriched reads in the antibody captured sample could be determined in an identity independent manner. In 11 out of the 13 patients, between 98% and 100% of the enriched analysis reads were of mammalian viral origin. In fecal samples between 21% and 100% viral reads were detected in the EAR in 4 out of 6 cases. Surprisingly, in two patients with enterovirus infection, no viral reads were detected in the EAR.

Of note are patient 1 and 2. For both patients viral reads were increased after capture (table 2), but the EAR contained no PIV-1 or PIV-2 sequences (table 3). This was caused by the comparison used to determine the EAR; reads which are present in the input are included in the analysis (see material and methods). Apparently the low number of viral reads in the captured material were from a different part of the viral genome than the few reads of the viral reads in the input. Therefore, it has to be kept in mind that using this approach during data analysis might diminish the chance to identify viruses which are hardly present in the input material when identification is only based on the detection of enriched sequences. On the other hand, inclusion of the reads which are only present in one of the two libraries might lead to wrong interpretations by the presence of experimental artefacts due to PCR errors and/or chimeric PCR products.

Also enteroviruses were poorly captured. A possible explanation might be that the bacteria or bacteriophage background in fecal samples is high which makes it more difficult to enrich a virus with a relatively low load. Nonetheless, we were able to reduce the amount of reads originating from bacteriophages and plant viruses (enrichment index of 0.01 and 0.08), indicating that this method can efficiently reduce the background in fecal samples. Furthermore, it could be that the immune response to enteroviruses is poor, especially in immunocompromised patients, which is the case in our study patients with diarrhoea. These patients were HIV-1 positive with relatively low CD4 counts (0.18 and 0.05×10E9 cells/L in the two patients with enterovirus infection). On the other hand, it has been published that HIV-1 infected patients are not hampered in their immune response to enteroviruses, and chronic shedding is as rare as in the normal population [25].

Antibody response to a virus infection can vary, depending on the virus, but also age and immune state of patients. Also, antibodies subclasses can differ; some viruses elicit a strong IgG response, while others induce a response dominated by IgA. In our antibody capture experiment, a combination of protein A and G coupled beads was used. Both proteins strongly bind to IgGs, but not or less efficient to IgA. It has been shown that serum antibodies, for instance against PIV-1 are not neutralizing efficiently, and that especially secretory IgA plays an important role in preventing re-infection [26], [27]. To address that point, protein L coupled beads, which are able to capture IgA, were tested. However, no difference was observed in the amount of PIV-1 or enterovirus capture (data not shown).

To date many new viruses have been identified [10]–[13], yet for a substantial number the link with disease remains to be established. Especially in stool samples, which contain many unknown viruses, it is important to determine whether novel viruses are pathogenic. A pathogenic virus elicits an immune response in the host, whereas bacteriophages and plant viruses, which are also massively present in stool, do not. The restriction that the agent has to be recognized by the patients’ antibodies adds an important selectivity tool to virus discovery when one wants to focus on pathogenic viruses.

In summary, a new selective approach for virus discovery is presented that enables detection of viruses that have recently elicited an immune response resulting in antibody production. The method appears to be an effective tool for reducing host DNA/RNA and bystander virus sequences and provides a selection for viruses that are able to elicit an antibody response, thus agents that are possibly pathogenic for the host. Moreover, the possibility of comparing reads obtained after antibody capture with reads obtained from the input samples is useful for identifying sequences not yet labelled in Genbank. Further analysis of those sequences (e.g. via genome walking) will allow the identification and characterization of viruses which are highly divergent from the currently known virus families.
